# Professional Commitment to Ethical Discussions Needed From Epidemiologists in the COVID-19 Pandemic

**DOI:** 10.2188/jea.JE20200278

**Published:** 2020-09-05

**Authors:** Kenji Matsui, Keiichiro Yamamoto, Yusuke Inoue

**Affiliations:** 1 Division of Bioethics and Healthcare Law, the National Cancer Center Japan, Tokyo, Japan; 2 Department of Biomedical Ethics, University of Tokyo Graduate School of Medicine, Tokyo, Japan; 3 Clinical Research Center, the National Center for Global Health and Medicine, Tokyo, Japan; 4 Department of Public Policy, University of Tokyo Institute of Medical Science, Tokyo, Japan

**Keywords:** COVID-19, epidemiology, ethics, journals, forum

The global threat due to the novel coronavirus 2019 infection (COVID-19) pandemic has given rise to various ethical challenges in public health, and increasingly more articles are being published in medical journals on ethical issues concerning COVID-19. One major theme concerns the moral distress of balancing the importance of epidemiological surveillance and digital patient tracing with privacy concerns.^1^^–^^4^ Given the extraordinary current circumstances, other pressing ethical questions have also been posed, including the following: Which studies should be continued or cancelled? What constitutes an ethical study design and rationale? How should the participant burden/risk be balanced against the benefits of research? Should protocol procedures be limited or modified to ensure participant safety, at the risk of decreasing research integrity? Should we justify a shortcut through a fast-track research review and an expedited submission-review process, with the potential downside of lower research quality and more faulty publications? How should the scientific community avoid jeopardizing the pandemic public health management by the rapid and uncontrolled flood of clinical/epidemiological data dissemination in the media? Should we justify conducting urgently required research in developing countries with more treatment-naïve potential research populations, even though the peak of infection expansion in developed countries has already waned?^5^^–^^17^

Conventionally, academic journals have been one of the most powerful venues for scholarly discussion. However, since the COVID-19 expansion, the extent to which scholarly journals of epidemiology have served as discussion forums for ethical issues on COVID-19 has become unclear, even though many of the ethical questions noted above would likely be important for epidemiologists as well. Against that backdrop, we conducted a small bibliometric survey on June 11, 2020. Specifically, we performed a database search of PubMed using the constraints of “COVID-19,” and “ethics.” This identified 424 articles, of which approximately two-thirds (285) were published in scholarly journals; the remaining 139 were in commercial journals. Of the former 424, 13 were published in Nature/Science, 33 in five major medical journals (*NEJM, BMJ, JAMA, Lancet, Annals of Internal Medicine*), 192 in other medical journals, 39 in public health/epidemiological journals, 64 in bioethics journals, and 36 in others. Of the 39 published in public health/epidemiological journals, only one was published in an epidemiological journal.^18^ Accordingly, our findings suggest that unfortunately, epidemiologist engagement in ethical issues on COVID-19 is minimal at best.

We speculate that this may be caused by the following factors: (1) Not all—but many—epidemiologists might be indifferent to ethical issues on epidemiology, or have left these arguments in the hands of others such as bioethicists; (2) While they may be concerned with ethical issues, epidemiologists may be less familiar with developing ethical arguments; (3) Appropriate forums for mediation of ethical discussions in epidemiological journals may be few in number. Notably, this third factor should be rejected because nearly all of the top 10 epidemiological journals with “epidmiol-” or the equivalent in their names, being indexed in the 2018 Journal Citation Reports^®^ (except *Journal of Epidemiology*, ranked 10^th^), provide sufficient space for unsolicited and unstructured longer articles (1,500–3,000 words) for development of debates or perspectives, in addition to the *Letter to the Editor* section. Therefore, we suspect that most epidemiologists are simply not using the available forums well. We believe that ethical issues directly concerning epidemiology should be shared and discussed, first and foremost, among epidemiologists. Because epidemiologists are health professionals, they shoulder the responsibility to maintain ethical integrity in their epidemiological endeavors. Utilizing such forums effectively and actively to exchange opinions on the ethics of epidemiology will promote the healthy development of epidemiology as a science and will contribute greatly to public health policymaking as well.^19^
